# Highly stable PtP alloy nanotube arrays as a catalyst for the oxygen reduction reaction in acidic medium†
†Electronic supplementary information (ESI) available. See DOI: 10.1039/c5sc00124b



**DOI:** 10.1039/c5sc00124b

**Published:** 2015-03-18

**Authors:** Lili Zhang, Meng Wei, Suqing Wang, Zhong Li, Liang-Xin Ding, Haihui Wang

**Affiliations:** a School of Chemistry & Chemical Engineering , South China University of Technology , Guangzhou , Guangdong 510640 , China . Email: lxding@scut.edu.cn ; Email: hhwang@scut.edu.cn

## Abstract

Nanostructured Pt-metal alloys have shown impressive catalytic properties for the oxygen reduction reaction (ORR) in acidic medium, but their long-term stability has not been satisfactory. Herein, we look beyond the traditional Pt-metal alloys and have developed a new kind of Pt-nonmetal alloy electrocatalyst for the ORR. Specifically, the novel catalyst is composed of interconnected platinum monophosphide (PtP) alloy nanocrystals (∼3–4 nm) and featured supportless nanotube array morphologies. Due to the unique combination of composition and structure, the obtained PtP alloy nanotube arrays not only exhibited remarkable ORR activity, but also showed almost no degradation of the half-wave potential after accelerated durability tests. The result suggests that alloying Pt with a nonmetallic element (such as P) is indeed an effective approach to address the poor stability of Pt-based catalysts in acidic medium.

## Abstract 2

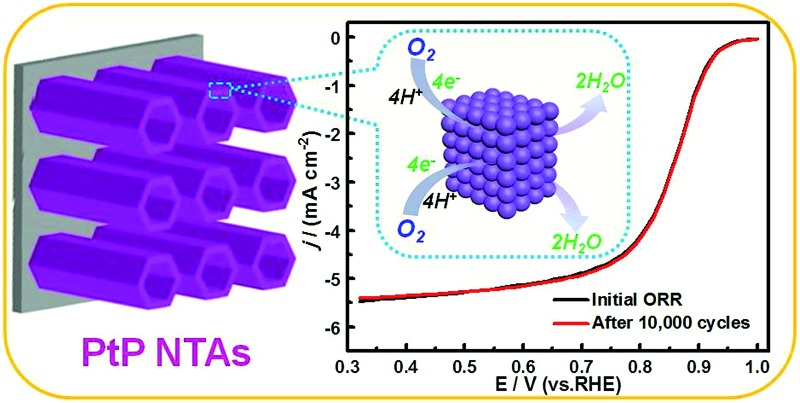
Self-supporting PtP nanotube arrays composed of interconnected PtP alloy nanocrystals exhibited excellent activity and durability for the ORR in acidic medium.

## Introduction

The sluggish kinetics of the oxygen reduction reaction (ORR) at the cathode is one of the major obstacles for the commercialization of proton exchange membrane fuel cells (PEMFCs).^1^ To overcome this barrier, great effort has been dedicated to developing highly active catalysts to improve the ORR performance over the past few decades.^2^ So far, platinum is still considered to be the most efficient catalyst to facilitate the ORR. However, a severe fact is that platinum as a scarce resource, therefore it is difficult to deploy in large-scale PEMFCs.^3,4^ Therefore, it is necessary to minimize the Pt loading, maximize the Pt utilization and extend the life time of Pt catalysts to meet the cost and performance requirements for PEMFC commercialization.

At present, alloying Pt with some less expensive transition metals along with a shape-controlled synthesis is the most widely used strategy to reduce the Pt loading without compromising catalytic activity.^3^ It has been demonstrated that Pt-based alloys with advanced nanostructures can achieve at least two to four times greater ORR activity compared to the commercial Pt/C catalysts due to the change in surface electronic structure and oxygen adsorption properties.^5^ However, although these Pt-based alloy catalysts have significantly reduced the Pt loading and shown remarkable activity for the ORR, several critical issues during PEMFC reactions, such as the dissolution of Pt or alloyed base metals, the poisoning of strongly adsorbed species, and the corrosion of carbon supports, could cause catalytic activity degradation, which still hinder their practical application in PEMFCs.

It is known that the durability problem of Pt-based catalysts can be alleviated by the use of a more corrosion-resistant catalyst structure or composition. For example, supportless one-dimensional (1D) Pt-based nanotubes/nanowires not only eliminate the support corrosion problem, but also make them less vulnerable to dissolution and Ostwald ripening than the zero-dimensional (0D) Pt nanoparticles.^6,7^ Zhang *et al.* employed Au clusters to stabilize the Pt ORR catalysts that showed a negligible loss of 4% in electrochemically active surface areas (ECSAs) after 30 000 cycles.^8^ Nevertheless, we found that the non-platinum components in these high stability Pt-based catalysts are mainly confined to the precious metals, such as Pd, Au, and so on. Other platinum-base metal alloy catalysts, even the current state-of-the-art PtCo and PtNi alloys^1a,4^ still inevitably suffer from dissolution/corrosion.

Recently, the combination of metals with some nonmetal elements has attracted much attention because nonmetal elements can significantly affect the physical and chemical properties of the metal material.^9–13^ A typical example is the use of Ni_2_P as a cocatalyst for Pd/C to form a Pd–Ni_2_P/C nano-electrocatalyst which not only shows remarkable catalytic activity, but also exhibits impressive corrosion resistance in acidic medium.^9^ In addition, Tang *et al.* demonstrated that alloying Pd with P can lead to a decrease in the adsorption strength and adsorption amount of CO.^10^ In this regard, alloying Pt with the nonmetallic element phosphorus seems to hold great promise to address the stability limitations of Pt-based catalysts for the ORR. Specifically, phosphorus should be an ideal candidate to replace the transition metals because of its smaller atomic radius, abundant valence electrons and excellent stability in both acid and alkali solutions. This idea, though interesting, is difficult to be achieve *via* a conventional method, due to the different properties between the platinum and nonmetallic phosphorus. In recent years, some studies have shown that phosphorus can be integrated into platinum-based alloys by a self-reduction method using Na_2_HPO_2_ or H_3_PO_2_, but the content of phosphorus in these platinum-based alloys is very low.^14^ More importantly, the reported preparation methods are difficult to further engineer the final morphology and composition. Up to now, phosphorus-containing catalysts are still only obtained in the form of nanoparticles and have no choice but to be supported on porous carbon materials, which still face a serious stability problem. Previous studies mostly focused on how to make use of the incorporative phosphorus to improve the catalytic activity. The strategy of using phosphorus to stabilize Pt metal electrodes for the ORR, especially in the form of a supportless architecture, has not been reported so far.

Herein, we introduce a facile electro-codeposition approach to controllably synthesize porous PtP alloy nanotube arrays (PtP NTAs) using ZnO nanorod arrays as a template. This proposed strategy not only successfully achieves the efficient introduction of elemental phosphorus, but also, compared with the conventional platinum-metal alloy catalysts, has the following prominent advantages: (i) the 1D self-supporting nanotube array architecture can effectively alleviate the many drawbacks of the 0D nanomaterials, such as carbon support corrosion, Ostwald ripening and nanoparticle aggregation *etc.*; (ii) the interconnected nanocrystals in the tube walls result in a porous structure, which can promote the transport of active species and improve the Pt utilization; (iii) as the transition metal replacement, alloying Pt with nonmetallic element phosphorus can avoid the dissolution or leaching of the base metals from the alloy surface; (iv) the addition of phosphorus can effectively modify the electronic state of the surface of the platinum atoms because of its abundant valence electrons. As expected, due to the above-mentioned advantages which originated from the integration of the composition and structure, the as-synthesized PtP NTAs exhibited excellent catalytic activity and durability for the ORR in acidic medium.

## Results and discussion

**Scheme 1:**
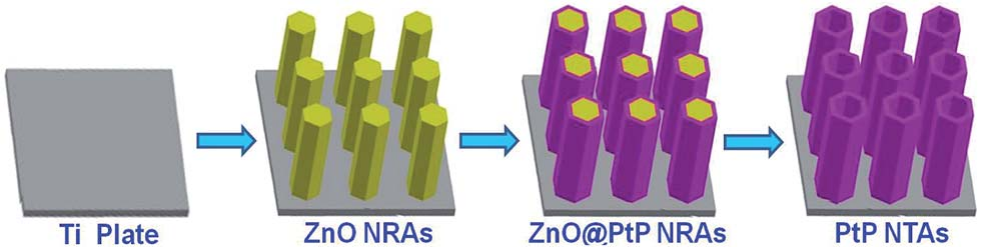
Schematic illustration for the synthesis of PtP alloy nanotube arrays.

**Fig. 1:**
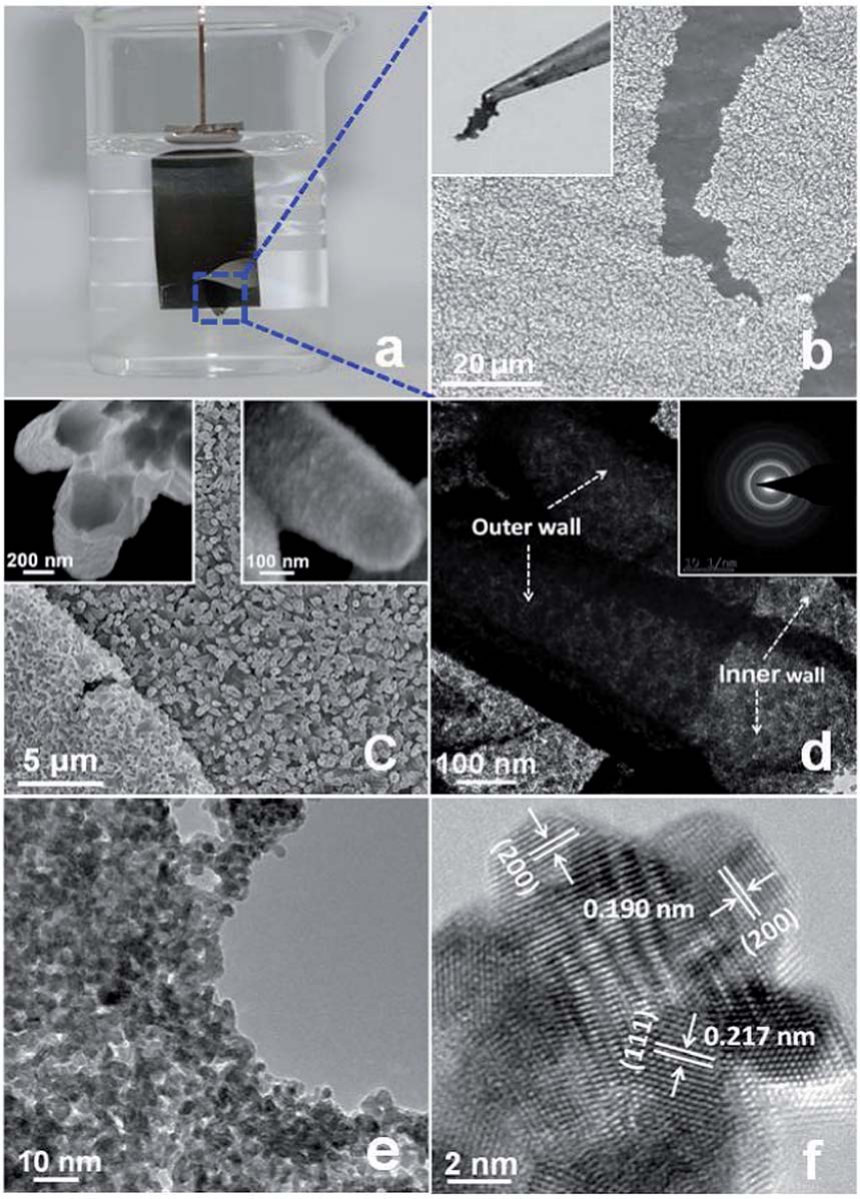
(a) Optical image of the PtP NTAs on the Ti substrate in H_2_SO_4_ solution (pH = 2.5). (b and c) SEM images and optical image (inset in (b)) of the PtP nanotube array sheet when peeled from the Ti substrate. The insets in (c) are high magnification SEM images. (d) TEM image and SAED pattern (inset in (d)), (e and f) HRTEM images of the PtP NTAs.

The PtP NTAs were fabricated *via* a simple two-step galvanostatic electrodeposition process, which is illustrated in Scheme 1. The optical image in Fig. 1a shows that these PtP alloy nanotubes are uniformly grown on the Ti substrate. The scanning electron microscopy (SEM) images in Fig. 1b and c show that these PtP alloy nanotubes are highly ordered and connect to each other to form a sheet. More interestingly, even if shed, they still perfectly kept their original sheet morphology (the inset in Fig. 1b), which allows us to easily move them onto a glassy carbon electrode for electrochemical tests. In addition, the triangle area in the lower left corner of Fig. 1c provides the bottom view of the overturned sample peeled from the Ti substrate. From the bottom view, a honeycomb like morphology can be clearly observed, indicating that the as-synthesized PtP alloy has a typical hollow nanotube array structure. Further magnified SEM images inset in Fig. 1c confirm that the PtP alloy nanotubes are connected with each other at the bottom and have a tube diameter of ∼350–450 nm, similar to the ZnO nanorod template (Fig. S1†). For comparison, the pure Pt NTAs were also synthesized in this study, as shown in Fig. S2,† by the same route.

Fig. 1d shows a typical transmission electron microscopy (TEM) image of the two adjacent PtP alloy nanotubes. It can be seen that the nanotube wall possesses a porous structure and the wall thickness is around 50 nm. Unlike other phosphorus-doped amorphous metal nanomaterials,^13^ the selected area electron diffraction (SAED) pattern, shown in the inset of Fig. 1d as diffraction rings, indicates that the as-synthesized PtP nanotubes are polycrystalline. The X-ray diffraction (XRD) analysis in Fig. S3† confirms that the PtP nanotubes are crystallized in the face-centered cubic (fcc) structure. A higher magnification TEM image in Fig. 1e shows that the PtP alloy nanotubes are porous and consist of homogeneous nanocrystals (∼3–4 nm). The structural details of the tube wall were further investigated using high resolution TEM, which is shown in Fig. 1f. The lattice fringes with interplanar spacings of 0.217 nm and 0.190 nm can be readily indexed to the (111) and (200) facets of Pt, respectively. It is worth mentioning that the interplanar spacing of the (111) and (200) facets in the PtP nanocrystals are slightly lower than bulk Pt (0.227 nm and 0.196 nm, JCPDS PDF#04-0802), revealing the contraction of the lattice by the incorporation of phosphorus in the fcc structure of Pt. Moreover, a set of obvious moiré fringes are observed in Fig. 1f, which should be due to the overlapping nanocrystals resulting in the superposition of crystalline lattices.^15^ Further detailed observation showed that the heterogeneous interfaces between these nanocrystals are indistinct and seem to be in the formation of a pseudo-alloy layer, suggesting that the PtP nanocrystals are closely connected at the atomic level at the direct contact interfaces, which will provide strong geometric and electronic effects to favor catalytic reactions.^16^

**Fig. 2:**
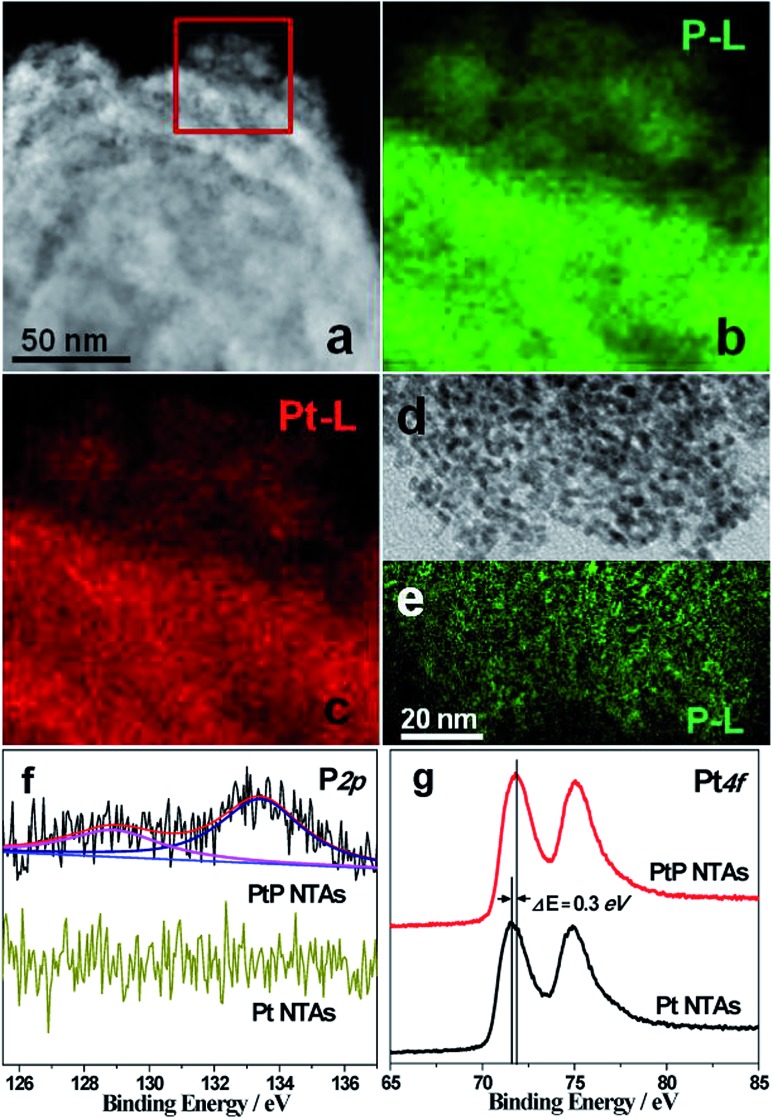
(a) HAADF-STEM image of PtP NTAs. (b and c) EELS mappings of P and Pt in the marked area in (a). (d) TEM image and (e) EELS mapping of P for the dispersed tube wall. (f and g) XPS spectra of P 2p and Pt 4f for PtP NTAs and Pt NTAs.

Fig. S4† shows the EDX pattern of the PtP NTAs. The strong spectral peaks of Pt and P confirm that these two elements underwent successful codeposition on the Ti plate substrate. Quantitative analysis results from ICP-AES measurements reveal a composition of ∼91 at.% Pt and ∼9 at.% P in the sample. Fig. 2a shows the HAADF-STEM image of a typical PtP alloy nanotube. Fig. 2b and c show the corresponding two-dimensional EELS mapping of enhanced P-L and Pt-L signals. The highly similar mappings indicate that the two elements are homogeneously distributed in the nanotube. Such elemental distributions also suggest a high quality alloy structure. Additionally, the EELS mapping for a dispersed tube wall was also performed. The green P-L (Fig. 2e) signals are highly consistent with the distribution of the nanoparticles (Fig. 2d), which further confirms high quality PtP alloy formation.

To further investigate the surface composition of the PtP NTAs, X-ray photoelectron spectroscopy (XPS) analyses were performed. As shown in Fig. 2f, two kinds of phosphorus species are observed on the surface of the PtP NTAs. The P 2p peak at 128.9 eV can be assigned to P^0^, which gives strong evidence for the existence of a PtP alloy. The peak at 133.4 eV can be assigned to oxidized phosphorus, which should be derived from the oxidation of the surface of the alloy nanoparticles. In addition, we have noticed that the lower binding energy peak at 128.9 eV shifted negatively by 1.5 eV from that of the pure phosphorus (130.4 eV). Meanwhile, a slight positive shift (0.3 eV) of the Pt 4f binding energy for the PtP NTAs compared with the pure Pt NTAs is also observed (Fig. 2g). The opposite shifts of the P 2p and Pt 4f binding energies reveal that there is a strong interaction between the Pt and P atoms, which should be due to the formation of the PtP alloy. Furthermore, the positive shift of the Pt 4f binding energy in the PtP NTAs can also be associated with a downshift d-band center,^17^ which can lead to the reduction of the oxygen adsorption energy.^18^ Obviously, this is exactly what is desired for an excellent ORR catalyst.

**Fig. 3:**
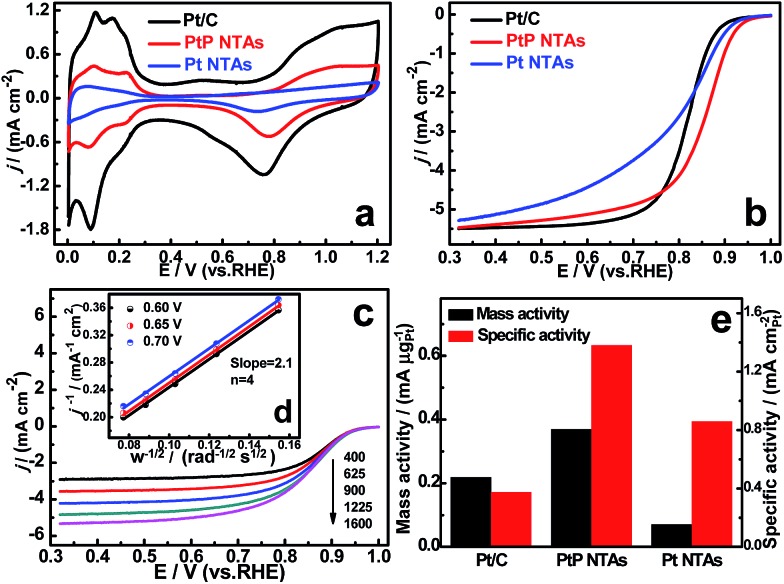
(a) Cyclic voltammetry curves for the PtP NTAs, Pt NTAs and Pt/C in N_2_-purged 0.5 M H_2_SO_4_ at a scan rate of 50 mV s^–1^ (Pt loading: ∼28 μg cm^–2^, S = 0.19625 cm_geo_^2^). (b) ORR polarization curves for the PtP NTAs (40 μg cm^–2^), Pt NTAs (112 μg cm^–2^) and Pt/C (28 μg cm^–2^) in O_2_-saturated 0.1 M HClO_4_ with a sweep rate of 5 mV s^–1^ and a rotation rate of 1600 rpm. (c) Rotation-rate-dependent current–potential curves for the PtP NTAs (40 μg cm^–2^) in O_2_-saturated 0.1 M HClO_4_. (d) Koutecky–Levich plots for the PtP NTAs at various potentials. (e) Comparison of mass activities and specific activities for the PtP NTAs, Pt NTAs and Pt/C at 0.85 V.

The ORR activities of the PtP NTAs were evaluated by cyclic voltammetry (CV) and rotating disk electrode (RDE) measurements. Here the sheets of PtP nanotube arrays were directly used for ORR measurements in this study due to their stable sheet-like structure. The pure Pt NTAs and a commercial Pt/C (20% Pt, Johnson Matthey) catalyst were tested for comparison. Cyclic voltammetry was initially performed on the three catalyst electrodes to measure their electrochemically active surface areas (ECSAs), and the corresponding cyclic voltammograms are shown in Fig. 3a. According to the method reported in the literature,^19^ the calculated ECSA of the PtP NTAs from the hydrogen desorption peaks is 26.7 m^2^ g_Pt_^–1^, which is ∼46% of the commercial Pt/C (58.2 m^2^ g_Pt_^–1^). The interconnected nanoparticles resulting in a large number of occupied interfaces are believed to be a key factor for the relatively lower ECSA of our PtP NTAs. In contrast, the pure Pt NTAs only exhibited a small ECSA of 8.1 m^2^ g_Pt_^–1^. This should be due to the obvious agglomeration in the tube wall (Fig. S2†). In addition, we also found that the oxidation and reduction peaks for the PtP NTAs were weaker and shifted to significantly more positive potentials compared to the commercial Pt/C, suggesting a low content formation or a weakening of the Pt-oxygenated species on the PtP NTAs. As mentioned by Viswanathan, this will be conducive to oxygen adsorption at low potential and thus promoting the ORR kinetics.^20^

Fig. 3b shows the typical ORR polarization curves of the three catalysts. It can be seen that the ORR onset and half-wave potentials of the PtP NTAs (0.96 V, 0.85 V) are significantly higher than that of the pure Pt NTAs (0.94 V, 0.80 V) and the commercial Pt/C catalyst (0.92 V, 0.82 V), indicating that the PtP NTAs have much lower overpotentials than the other two catalysts. Currently, it is widely accepted that the breaking of the O–O bond is a rate-determining step in the ORR, which is generally affected by the Pt d-band center.^18^ By combining the XPS measurement results, we are convinced that the improved ORR activity of the PtP NTAs is mainly ascribed to the incorporation of phosphorus resulting in a reduction of the oxygen adsorption energy because of the downshift Pt d-band center. In addition, we found that the Pt NTA catalyst also displays a more positive onset potential than the commercial Pt/C catalyst. This result indicates that the nanotube architecture is also an important reason for the promotion of the oxygen reduction activity, which should be due to the interconnected Pt nanocrystals resulting in an enhanced interface effect. In addition, we further observed that the slope of the polarization curve of the Pt NTAs is significantly less than the commercial Pt/C and PtP NTA catalysts, showing almost no limiting-current. This unusual result is most likely due to the intimate aggregation of the Pt nanoparticles, which results in slow oxygen diffusion in the Pt NTA catalyst layer. To further evaluate the kinetic parameters for the ORR on the PtP NTAs, the rotation-rate-dependent polarization curves were recorded and are shown in Fig. 3c. The corresponding transferred electron number (*n*) evaluated from the Koutecky–Levich (K–L) plots is calculated to be 4 at 0.60–0.70 V (Fig. 3d), showing an almost complete four-electron oxygen reduction process on the PtP NTAs. Even more interesting is that, despite having a reduced Pt ECSA, the PtP NTAs exhibit much higher mass and area-specific activities than the pure Pt NTAs and Pt/C catalysts for the ORR at 0.85 V (Fig. 3e), which is similar to the reported free-standing Pt-nanowire membrane and supportless PtPd nanotubes.^6,7^

**Fig. 4:**
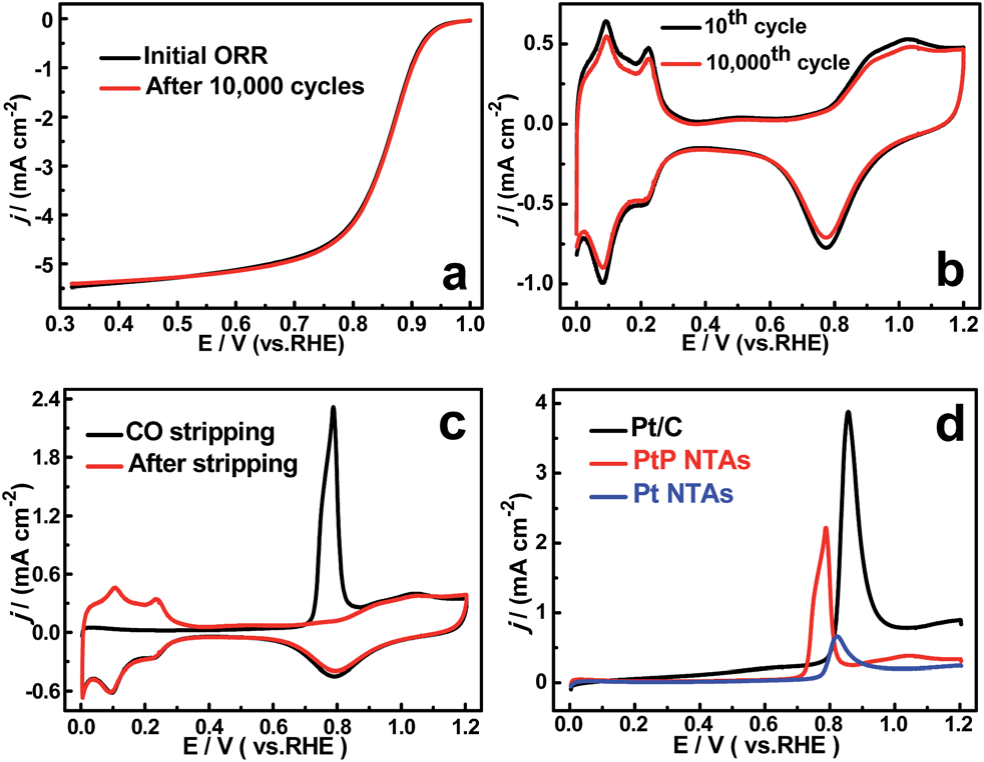
(a) ORR polarization curves before and after 10 000 potential cycles between 0.6 V and 1.0 V for the PtP NTAs in O_2_-saturated 0.1 M HClO_4_. (b) Cyclic voltammetry curves for the 10th and 10 000th cycles of the PtP NTAs (40 μg cm^–2^) in N_2_-purged 0.5 M H_2_SO_4_. (c) CO stripping voltammograms of the PtP NTAs performed in 0.5 M H_2_SO_4_ solution at 50 mv s^–1^. (d) The comparison of CO stripping voltammograms of the PtP NTAs, Pt NTAs, and Pt/C (JM) (Pt loading: ∼28 μg cm^–2^).

To confirm the enhanced stability of the PtP NTAs for the ORR, an accelerated durability test was performed by continuously linear potential sweeps between 0.6 V and 1.0 V at a scan rate of 50 mV s^–1^ in an O_2_-saturated HClO_4_ solution. As shown in Fig. 4a, identical polarization curves with almost no degradation in the half-wave potential were observed for the PtP NTA catalyst electrode after 10 000 potential cycles, whereas there is about 100 mV and 28 mV degradation for the pure Pt NTAs (Fig. S6a†) and commercial Pt/C catalyst (Fig. S6b†), respectively. These comparisons clearly demonstrate that alloying Pt with phosphorus is indeed an effective approach to improve its cycling stability for the ORR. Moreover, considering the retention of the ECSA is an important parameter for a spent electrocatalyst, the cyclic voltammetry curves of the three catalysts before and after potential cycling were also recorded, which are shown in Fig. 4b and S6.† It can be clearly seen that the peak current densities in the hydrogen desorption regions (0.0–0.35 V) for the pure Pt NTAs (Fig. S6c†) and commercial Pt/C (Fig. S6d†) dropped dramatically after 10 000 potential cycles; in contrast, the PtP NTAs only exhibited a slight change (Fig. 4b). Further calculations show that the PtP NTAs lose ∼9% of their ECSA, which is significantly lower than that of the pure Pt NTAs (31%) and commercial Pt/C catalyst (38.7%). It has been demonstrated that the loss of the Pt ECSA is primarily due to the dissolution of Pt surface atoms or agglomeration of Pt nanoparticles through surface oxidation/reduction processes.^6^ Herein, the lower ECSA loss strongly suggests that the PtP NTAs have excellent structural stability, which further confirms the stabilizing effect of nonmetallic phosphorus in the PtP NTAs. It is worth noting that, in addition to the formation of a stable alloy structure, the preferred four-electron reduction process should also be an important contributing factor to the high stability of the PtP NTAs, which effectively prevents the formation of peroxide intermediates and accordingly diminishes the probability of Pt corrosion.^4^

In addition to the high cycling and structure stability, the PtP NTAs also exhibited a remarkable anti-poison capability for strongly adsorbed species, which was confirmed by performing CO stripping studies, as shown in Fig. 4c and d. It can clearly be seen that the onset potential (0.71 V) of CO oxidation on the PtP NTAs is obviously more negative than that on the Pt NTAs (0.77 V) and commercial Pt/C (0.79 V). The comparative result indicates that the presence of phosphorus can facilitate the removal of CO from the surface of the PtP NTAs, and thus confirms the enhanced CO-tolerant performance. For the same reason as the improved ORR activity, the enhanced CO tolerance ability of the PtP NTAs could be due to the geometric and electronic changes which originate from the incorporation of nonmetallic phosphorus. The strong correlation between the Pt d-band center and the adsorption energy of CO has been fully proven through a theoretical study by Nørskov and co-workers.^21^

## Conclusions

In summary, the nonmetallic-incorporated PtP NTAs composed of interconnected nanocrystals were successfully synthesized *via* a facile electrodeposition method. Compared with the pure Pt nanotube arrays and the commercial Pt/C, the PtP NTAs exhibit a significantly enhanced electrocatalytic activity and stability for the ORR in an acidic medium. Particularly, the catalyst showed almost no degradation in ORR activity after 10 000 potential cycles. It proves that alloying Pt with a nonmetallic element (such as P) is indeed an effective approach to improve the stability of Pt in acidic medium. The superior electrocatalytic performances can be mainly ascribed to the enhanced geometric and electronic effects and the stable PtP alloy structure, as well as the unique combination of dimensions. This study provides a new strategy for designing efficient Pt-based oxygen reduction catalysts with both excellent electrocatalytic activity and durability.

## Experimental section

### Synthesis of PtP and Pt nanotube arrays

All chemical reagents were analytical (AR) grade. Galvanostatic electrodeposition was achieved in a simple two-electrode electrolytic cell and the graphite electrode was used as a counter electrode (spectral grade, 1.8 cm^2^). Both PtP nanotube arrays and Pt nanotube arrays were synthesised by a template-assisted electrodeposition method.^22^ First, a ZnO nanorod array (NRA) template was electrodeposited on the Ti plates (99.99%, 2.5 × 1.0 cm) in 0.01 M Zn(NO_3_)_2_ + 0.05 M NH_4_NO_3_ solutions with a current density of 0.5 mA cm^–2^ at 70 °C for 90 min. Second, ZnO@PtP nanorod arrays were prepared by electrodepositing PtP nanocrystals on the surfaces of the ZnO NRAs in a solution of 1.16 × 10^–3^ M H_2_PtCl_6_ + 2.84 × 10^–3^ M NaH_2_PO_2_ + 3.4 × 10^–4^ M C_6_H_5_Na_3_O_7_·2H_2_O (the pH value was controlled to 3.5 with NaOH) with a current density of 0.25 mA cm^–2^ at 30 °C for 20 min, and ZnO@Pt nanorods were deposited under the same conditions without NaH_2_PO_2_. Then, PtP and Pt nanotube arrays were obtained by immersing ZnO@PtP and ZnO@Pt nanotube arrays in H_2_SO_4_ solution (pH = 2.5) for 5 h.

### Characterization of the catalysts

The structure and morphology were characterized by X-ray diffraction (XRD, Bruker D8 Advance), scanning electron microscopy (SEM, NOVA NANOSEM 430), energy dispersive X-ray spectroscopy (EDS, INCA 300), transmission electron microscopy (TEM, JEM-2010HR), high-angle annular dark-field scanning TEM (HAADF-STEM, FEI Tecnai G2 F30) and X-ray photoelectron spectroscopy (XPS, ESCALAB 250). All XPS spectra were corrected using the C 1s line at 284.6 eV. Curve fitting and background subtraction were accomplished.

### Electrochemical measurements

All the electrochemical measurements were carried out using an electrochemical workstation (IM6ex) with a standard three-electrode electrolytic cell, where a platinum wire was used as a counter electrode and a SCE was the reference electrode. A glassy carbon (GC) disk with a diameter of 5 mm served as the substrate for the working electrode. Prior to use, the GC disk was polished using alumina suspensions (0.05 μm Al_2_O_3_) on a felt polishing pad. After ultrasonic cleaning, a small piece of as-prepared PtP nanotube array film (∼8 μg) was adhered on the GC disk using Nafion solution (4–5 μL, 0.05 wt%). The details for the electrochemical measurements are included in the ESI.† In the calculation process, the potential should be + 0.244 V in 0.5 M H_2_SO_4_ and + 0.303 V in 0.1 M HClO_4_ *versus* the reversible hydrogen electrode (RHE).
