# Supplementary material for: Highly stable PtP alloy nanotube arrays as a catalyst for the oxygen reduction reaction in acidic medium
Source: Chem Sci. 2015 Mar 18;6(5):3211–6. doi: 10.1039/c5sc00124b (PMC5490421; doi:10.1039/c5sc00124b)
Supplement: Supplementary file 1 [file SC-006-C5SC00124B-s001.pdf]

Electronic Supplementary Material (ESI) for Chemical Science.
This journal is © The Royal Society of Chemistry 2015

Supporting Information

Highly Stable PtP Alloy Nanotube Arrays Catalyst for Oxygen Reduction Reaction in Acidic Medium**

Lili Zhang, Meng Wei, Suqing Wang, Zhong Li, Liang-Xin Ding,* and Haihui Wang*

Electrochemical measurements:

(1) Cyclic voltammetry (CV) measurements. CV measurements were conducted in N_2 saturated 0.5 M H_2SO_4 solution at a scan rate of 50 mV s^{-1}. The electrochemically active surface area (ECSA) of catalysts was calculated using eq.1 and eq.2:[1]

$$\mathrm{ECSA} = Q_H/(m \times q_H) \qquad (1)$$

Where Q_H is the under-potentially deposited hydrogen charge, m is the loading amount of metal, and q_H is the charge required for monolayer adsorption of hydrogen on a Pt surface, which is assumed to be 0.21 mC cm^{-2}.

$$Q_H = \int_0^{0.35} \frac{I[A] \times dE[V]}{v[V/S]} \qquad (2)$$

Where I is the current, E the potential, v the scan rate.

(2) Oxygen Reduction Reaction (ORR). ORR were test in O_2 saturated 0.1 M $HClO_4$ solution at 5 mV s^{-1}, and the scans rate of stability measurements were 50 mV s^{-1} between 0.6 V and 1.0 V. The transfer electron numbers per oxygen molecule in the ORR was determined by the K-L equations as follows:[2]

$$\frac{1}{j} = \frac{1}{j_k} + \frac{1}{Bw^{1/2}} \qquad (3)$$

where j is the measured current density, j_k is the kinetic current density and w is the angular velocity of the disk (w=2πN, N is the linear rotation speed).

$$B = 0.62nFC_{O_2}(D_{O_2})^{2/3}v^{-1/6} \qquad (4)$$

where n represents the overall number of electrons gained per oxygen, F is the Faraday

constant (F=96485 C mol^{-1}), C_{O2} is the bulk concentration of O_2 (1.26×10^{-3} mol L^{-1}), D_{O2} is the diffusion coefficient of O_2 in 0.1 M $HClO_4$ electrolyte (1.93×10^{-5} cm^2 s^{-1}), v is the kinetic viscosity of the electrolyte (1.009×10^{-2} cm^2 s^{-1}).

(3) CO stripping voltammetrys. The CO stripping measurements were also finished in 0.5 M H_2SO_4 solution. After the solution was purged with pure N_2 gas for 20 min, high purity CO was bubbled into 0.5 M H_2SO_4 electrolyte solution at 0 V for 15 min. Then, remove the remaining CO by bubbling pure N_2 gas into the electrolyte for 30 min, and continuous CVs were measured at a scan rate of 50 mV s^{-1}, where the first one was the CO stripping process.

Results and discussion

The low magnification SEM image in Figure S2a show that the pure platinum nanotubes are uniformly grown on titanium plate substrate and possess obvious array morphology, which are highly similar to the PtP NTAs. The magnified SEM image in Figure S2b shows that the surface of pure Pt nanotubes is rougher than the PtP alloy nanotubes, especially at the top. This may be due to the absence of phosphorus resulting in the uneven deposition of Pt on the ZnO nanorod surface. The typical hollow tube projection in Figure S2c clearly confirms the successful synthesis of pure platinum nanotube arrays. Moreover, a careful inspection of the structure of the pure platinum nanotube in Figure S2c indicated that there are many dark dots dispersed onto the surface of nanotubes, suggesting that these nanotubes have a rough tube wall structure. The observation is consistent with the SEM images mentioned above. A higher magnification TEM image in Figure S2d reveals that the pure platinum nanotubes are consists of the homogeneous overlapped nanocrystals, similar to the PtP NTAs. The structural details of the pure platinum nanotubes were further investigated by high resolution TEM, and the results are shown in the Figure S2e-f. It can be clearly seen that the Pt nanocrystals are closely connected each other without obvious interface, especially in the raised portion of the tube-wall surface (Figure S2f). This result indicated that the addition of phosphorus not only

as an alloying constituent, and can significantly affect the microstructure of the nanotubes.

References

1 J. N. Tiwari, K. C. Kemp, K. Nath, R. N. Tiwari, H.-G. Nam, K. S. Kim, *ACS Nano*, 2013, **7**, 9223.

2 (a) M. Shao, T. Huang, P. Liu, J. Zhang, K. Sasaki, M. Vukmirovic, R. Adzic, *Langmuir*, 2006, **22**, 10409; (b) V. Stamenković, T. J. Schmidt, P. N. Ross, N. M. Marković, *J.Electroanal. Chem.*, 2003, **554**, 191.

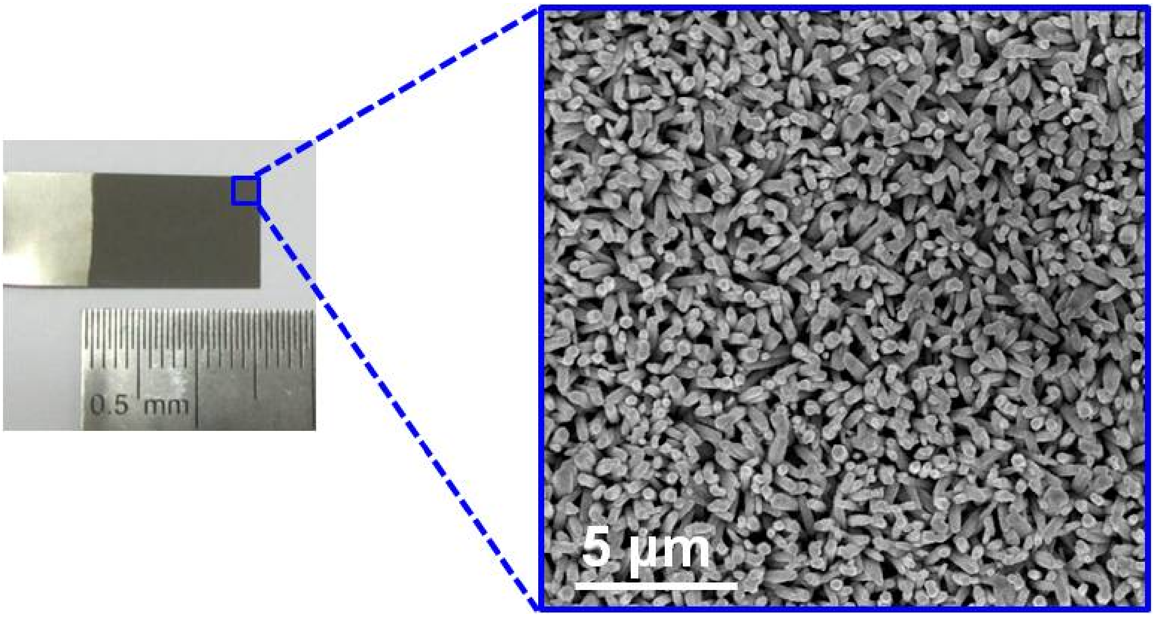

Figure S1. SEM image of the ZnO nanorad arrays.

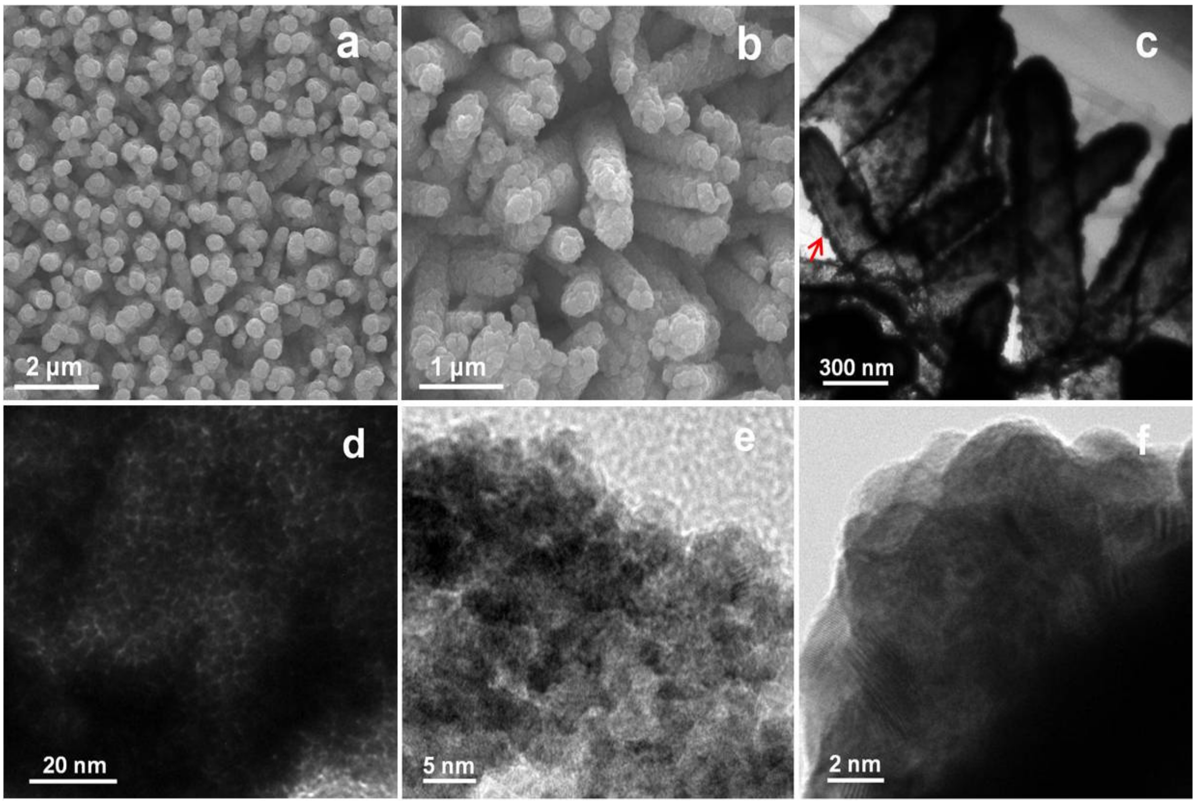

Figure S2. (a-b) SEM images of the Pt nanotube arrays. (c) TEM images of the Pt nanotube arrays. (d-e) HRTEM images of the Pt nanotube. (f) HRTEM images of the red arrow points part in (c).

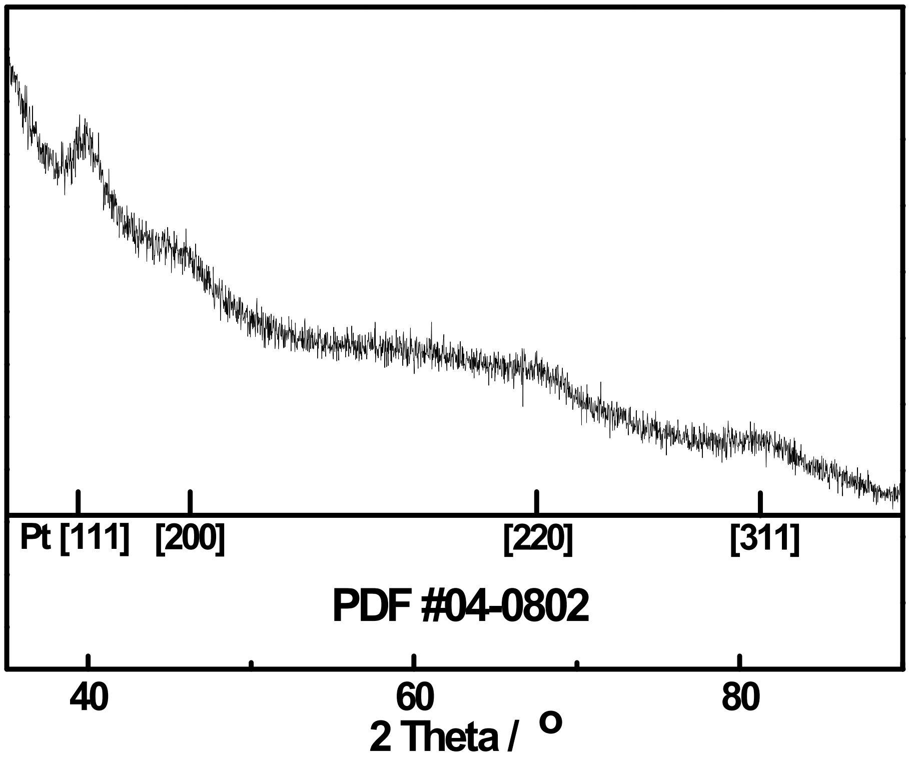

Figure S3. XRD pattern of the PtP nanotube arrays.

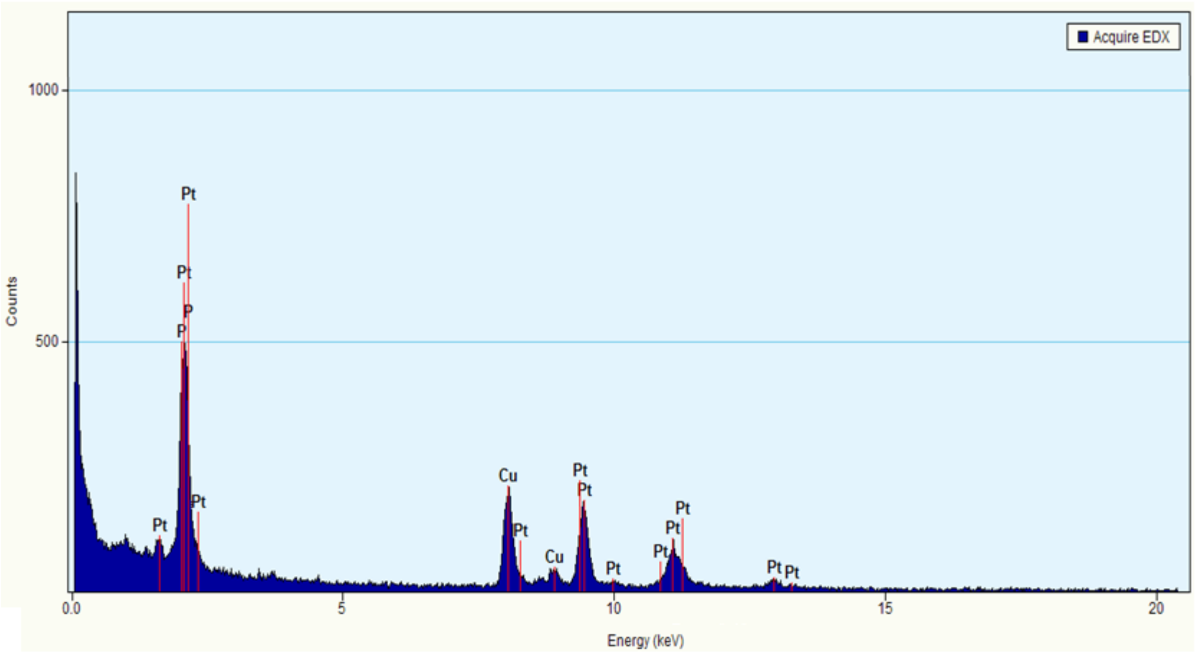

Figure S4. EDX profile of the PtP nanotube arrays.

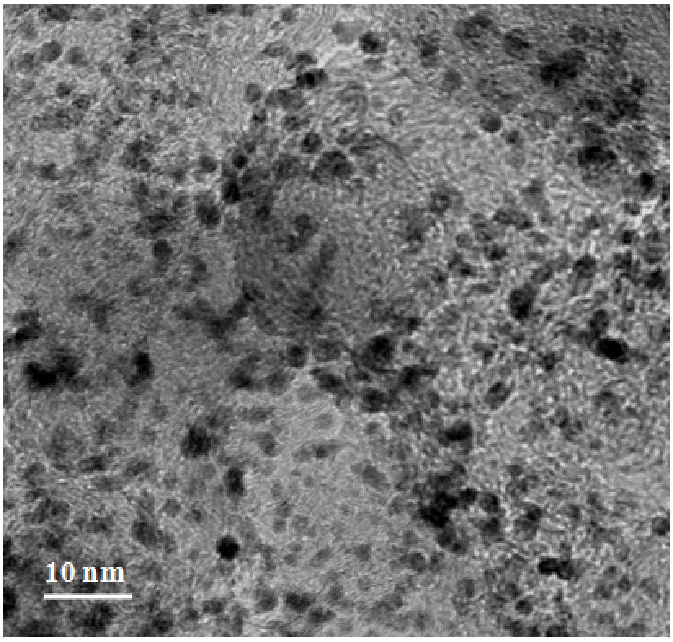

Figure S5. TEM image of the commerial Pt/C (JM).

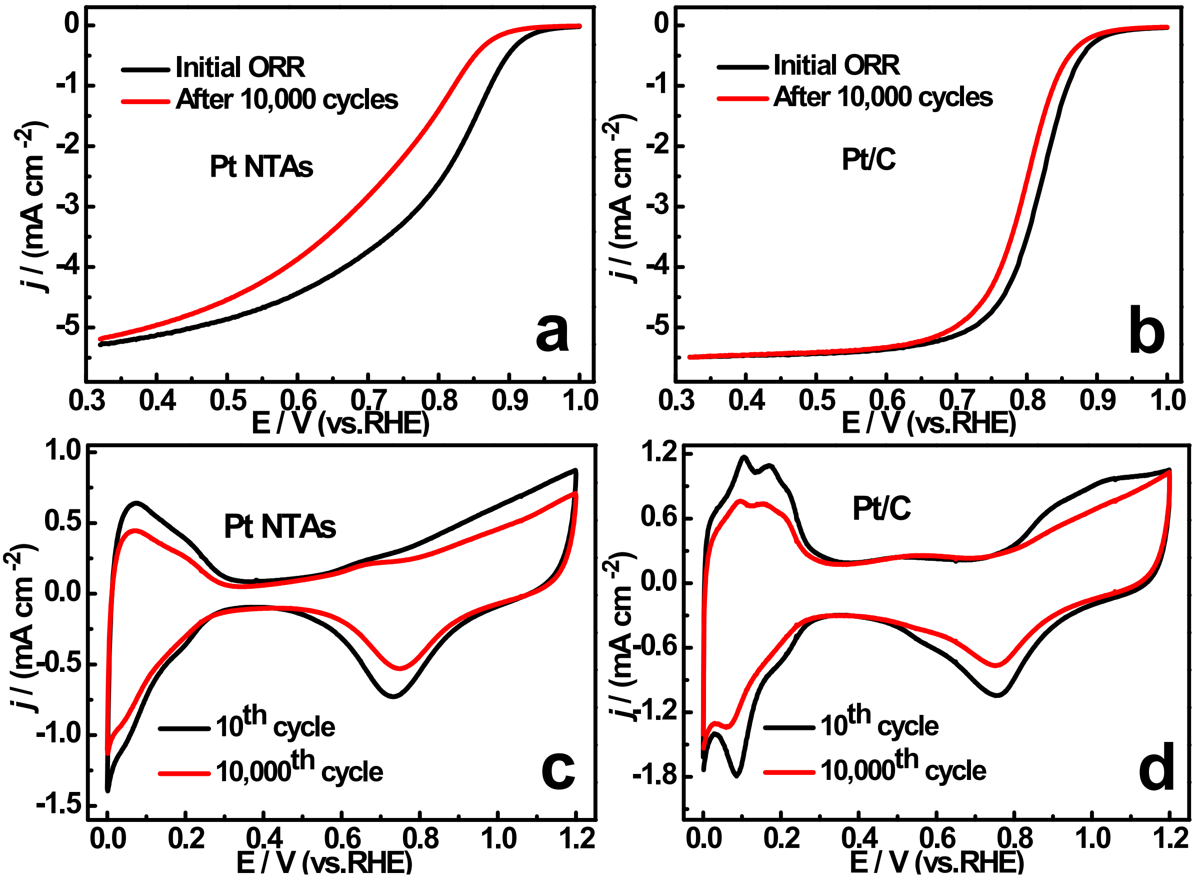

Figure S6. ORR polarization curves before and after 10,000 potential cycles between 0.6 V and 1.0 V for (a) Pt NTAs, and (b) Pt/C (JM) in O_2-saturated 0.1 M $HClO_4$. Cyclic voltammogram curves for the 10th and 10,000th cycles of (c) Pt NTAs, and (d) Pt/C (JM) in N_2-purged 0.5 M H_2SO_4 (Pt loading for Pt NTAs, commercial Pt/C are 112 μg cm^{-2}, 28 μg cm^{-2}, respectively.).
